# Supplementary material for: Patterns of Intron Gain and Loss in Fungi
Source: PLoS Biol. 2004 Nov 30;2(12):e422. doi: 10.1371/journal.pbio.0020422 (PMC532390; doi:10.1371/journal.pbio.0020422)
Supplement: Table S1 — Also available at http://genes.mit.edu/NielsenEtAl/. (4.3 MB ZIP). [file pbio.0020422.st001.zip › NielsenEtAl/html/706.html]

AN7665.1.NCU03268.1.MG09936.1.FG01091.1


```
 CLUSTAL W (1.82) Multiple Sequence Alignments - Introns Inserted


Sequence 1: NCU03268.1	251 aa
Sequence 2: MG09936.1	260 aa
Sequence 3: FG01091.1	250 aa
Sequence 4: AN7665.1	248 aa
Alignment Length: 268 aa
Number Identitical Residues: 68 aa
Alignment Score (without introns) 4053


MG09936.1 	MPSRGEPAALVSHIPRADGSAIYS--HAGYTVTASANGPIEAQ-RRDED-PYEALVDVIV
NCU03268.1	MATVT-PEASLNALPRADGSARYS--YAGYTVTASVNGPIEAQ-RRDEH-AYEAHVDVIV
FG01091.1 	MAPFTEPTAEISLLAKSDGSATFS--YGGYAVIAAVNGPVEAQ-RRDEN-AFEALVDVIV
AN7665.1  	---MAGPVADLHPLSRSDGSASYKCPFTGSNILGSVNAPIELPGRRDALKPEEATIEVFV
          	      * * :  :.::**** :.... *  : .:.*.*:*  .***  .. ** ::*:*

MG09936.1 	RPAAGVGG1TRERHLESLLQATLRQIILVKN--FPRGLIQVVLQVKSSPENDYASTKLVQ
NCU03268.1	RPSAGVGG1TRERHLEFILQSSLSQIILVKN--FPRSVIQIVLQVESTPENAYVNTKLVQ
FG01091.1 	RPAAGVGG1TRERQLESIMQAALRQLIPVRD--YPRSVIQITLQVAETPENAYVNAKIVQ
AN7665.1  	KPGTAPGG~VGERYVEGIVRSALGRVILGREKGYPRRGVVITLAIVG-------GEGVAR
          	:*.:. ** . ** :* :::::* ::*  ::..:**  : :.* :         .  :.:

MG09936.1 	ANL0NLPIIPALLQAAVLALLSAAIPLRATATCTVIAVVAGSDGEAESNLILDPSPREAE
NCU03268.1	ASL0NLPIIPALFQTAVLALLSAAVPMKATATSTVVAVVS-DDG--SKKIVADPSPQDIR
FG01091.1 	AQL0NLPIIPALLHSAILGLLSAAIPLKTIGAATLIAVPE-EEG---KDIIVDPSAVDID
AN7665.1  	GGS0YLPLLPALLHTATLALLSASVPLSVTLSATILAVDP--AG----KIIREPSTKEAK
          	.    **::***:::* *.****::*: .  :.*::**     *    .:: :**. :  

MG09936.1 	GARSVHALAFTSGDRLILAESEGHFTMQEWADVHAKALKICCEPVRDPGNNDMDMGDDNS
NCU03268.1	EATSLHVLAFTSHDELLLSESEGDFTVKEWDGVYENAQKICCQTASKQDGIDMVLDDDAS
FG01091.1 	RAKSVHALAFTSHDELLLSESEGAFTPDEWTKVLQLGQRVCCEHQ-QPGLDTAMSGDDIE
AN7665.1  	AAASLHVLAFTSKGHLLLNESEGAFTYDTWEAVYERALVICLGSSALSSDGDVAMAESTE
          	 * *:*.***** ..*:* **** ** . *  *   .  :*       .       :. .

MG09936.1 	TDVEGPNMRHFIRETMAAKVSADLHWKG--
NCU03268.1	S----PDMRHFLRSTLETKVASDLHWK---
FG01091.1 	S----PSMRQFIRSAMGTKVAEDLYWK---
AN7665.1  	S----QPLEGILRDTVEDHIHSEYSWKLAA
          	:      :. ::*.::  ::  :  ** ::
```
